# Understanding etiology of community-acquired central nervous system infections using metagenomic next-generation sequencing

**DOI:** 10.3389/fcimb.2022.979086

**Published:** 2022-09-26

**Authors:** Shanshan Zhang, Gang Wu, Yuru Shi, Ting Liu, Liangfei Xu, Yuanyuan Dai, Wenjiao Chang, Xiaoling Ma

**Affiliations:** ^1^ Department of Medical Oncology, The First Affiliated Hospital of University of Science and Technology of China (USTC), Division of Life Sciences and Medicine, University of Science and Technology of China, Hefei, China; ^2^ Department of Clinical Laboratory, The First Affiliated Hospital of University of Science and Technology of China (USTC), Division of Life Sciences and Medicine, University of Science and Technology of China, Hefei, China

**Keywords:** community-acquired central nervous system infections, etiology, metagenomic next-generation sequencing, diagnosis, cerebrospinal fluid

## Abstract

**Background:**

Community-acquired central nervous system infections (CA-CNS infections) have the characteristics of acute onset and rapid progression, and are associated with high levels of morbidity and mortality worldwide. However, there have been only limited studies on the etiology of this infections. Here, metagenomic next-generation sequencing (mNGS), a comprehensive diagnosis method, facilitated us to better understand the etiology of CA-CNS infections.

**Methods:**

We conducted a single-center retrospective study between September 2018 and July 2021 in which 606 cerebrospinal fluid (CSF) samples were collected from suspected CNS infectious patients for mNGS testing, and all positive samples were included in this analysis

**Results:**

After the exclusion criteria, a total of 131 mNGS-positive samples were finally enrolled. Bacterial, viral, fungal, parasitic, specific pathogen and mixed infections were accounted for 32.82% (43/131), 13.74% (18/131), 0.76% (1/131), 2.29% (3/131) and 6.87% (9/131), respectively. A total of 41 different pathogens were identified, including 16 bacteria, 12 viruses, 10 fungi, and 1 parasite and 3 specific pathogens. The most frequent infecting pathogens are *Epstein-Barr virus* (n = 14), *Herpes simplex virus 1* (n = 14), *Mycobacterium tuberculosis* (n = 13), *Streptococcus pneumoniae* (n = 13), and *Cryptococcus neoformans* (n = 8). Some difficult-to-diagnose pathogen infections were also detected by mNGS, such as *Streptococcus suis*, *Pseudorabies virus*, *Bunyavirus*, *Orientia tsutsugamushi* and *Toxoplasma gondii*.

**Conclusion:**

In this study, mNGS identified a wide variety of pathogens of CA-CNS infections and many of which could not be detected by conventional methods. Our data provide a better understanding of the etiology of CA-CNS infections and show that mNGS represents a comparative screening of CSF in an unbiased manner for a broad range of human pathogens.

## Introduction

Central nervous system (CNS) infections are inflammations of the brain and spinal cord caused by pathogenic microbes. Various environmental or commensal microorganisms can migrate through the blood-brain barrier (BBB) into the CNS to cause inflammation, manifested as fever, headache, vomiting, neck stiffness, disturbance of consciousness, convulsions and focal neurological deficits (Granerod et al., 2010). Although the incidences of CNS infections have fallen to 0.9 per 100,000 population in high-income countries like the USA, the global number of reported cases has risen during the past decade, particularly in Africa, with incidences up to 1000 per 100,000 population (GBD 2016 Neurology Collaborators, 2018). Despite the existence of antibiotic therapies, CNS infections are devastating clinical conditions that cause substantial mortality and severe sequelae (Brouwer et al., 2010).

Pathogen detection is important for diagnosis and treatment of CNS infections. Conventional methods used in clinical microbiology laboratories include culture techniques, antigen tests and polymerase chain reaction (PCR) (Brand et al., 2016). Culture-dependent method is regarded as the gold standard for pathogen diagnosis of infectious diseases, but this approach may take several days, and the results may be negative if antimicrobials are administered empirically or if the infection is caused by fastidious or nonculturable microorganisms like viruses (Brand et al., 2016). Antigen tests have a limited variety of reagents and are used only for the diagnosis of a few pathogen infections, such as Cryptococcus (Antinori et al., 2005). Although multiplex PCR assays, such as FilmArray meningitis or encephalitis panel are considered sensitive methods that allow the rapid detection and identification of infectious agents, they are restricted to a partial number of suspected microorganisms, limiting their utility as a standalone test in CNS infections diagnostics (Leber et al., 2016).

Recently, metagenomic next-generation sequencing (mNGS) has emerged as an unbiased technology that allows the simultaneous identification of all microorganisms in a single clinical sample (Goldberg et al., 2015; Miao et al., 2018; Miller et al., 2019). This culture-independent application ensures a detailed sequencing of the total DNA or RNA content of the microbiome and can be used for universal pathogen detection regardless of the microbe type (e.g., bacteria, viruses, fungi or parasites) (Schlaberg et al., 2017). Owing to its sensitivity, speed, and cost-effectiveness considerations, mNGS has the potential to become a routine workup in infectious disease diagnosis and pathogen identification of unknown origin.

CNS infections can be acquired spontaneously in the community (community-acquired CNS infections [CA-CNS infections]) or in the hospital as a complication of invasive procedures or head trauma (hospital-acquired CNS infections [HA-CNS infections]) (van de Beek et al., 2006; van de Beek et al., 2010). Compared with HA-CNS infections, CA-CNS infections are caused by a wider variety of pathogens, including bacteria, viruses, fungi, parasites and other specific microbes (van de Beek et al., 2021). Because of the limitations of conventional diagnostic methods for CNS infections, most of the current data on epidemiology of CNS infectious syndromes were focused on specific types of pathogens (van Kassel et al., 2020; van de Beek et al., 2021). In addition, vaccination and epidemiology make the etiology of CA-CNS infections differ among different countries and regions, and there is still a lack of studies to facilitate our investigation on etiology of CA-CNS infections. Therefore, we performed a retrospective study of a large-scale cohort that was positive for CSF pathogen detection using mNGS, and attempted to provide a better understanding of etiology of CNS infection in community settings via one standalone detection method.

## Materials and methods

### Study design

**Figure 1:**
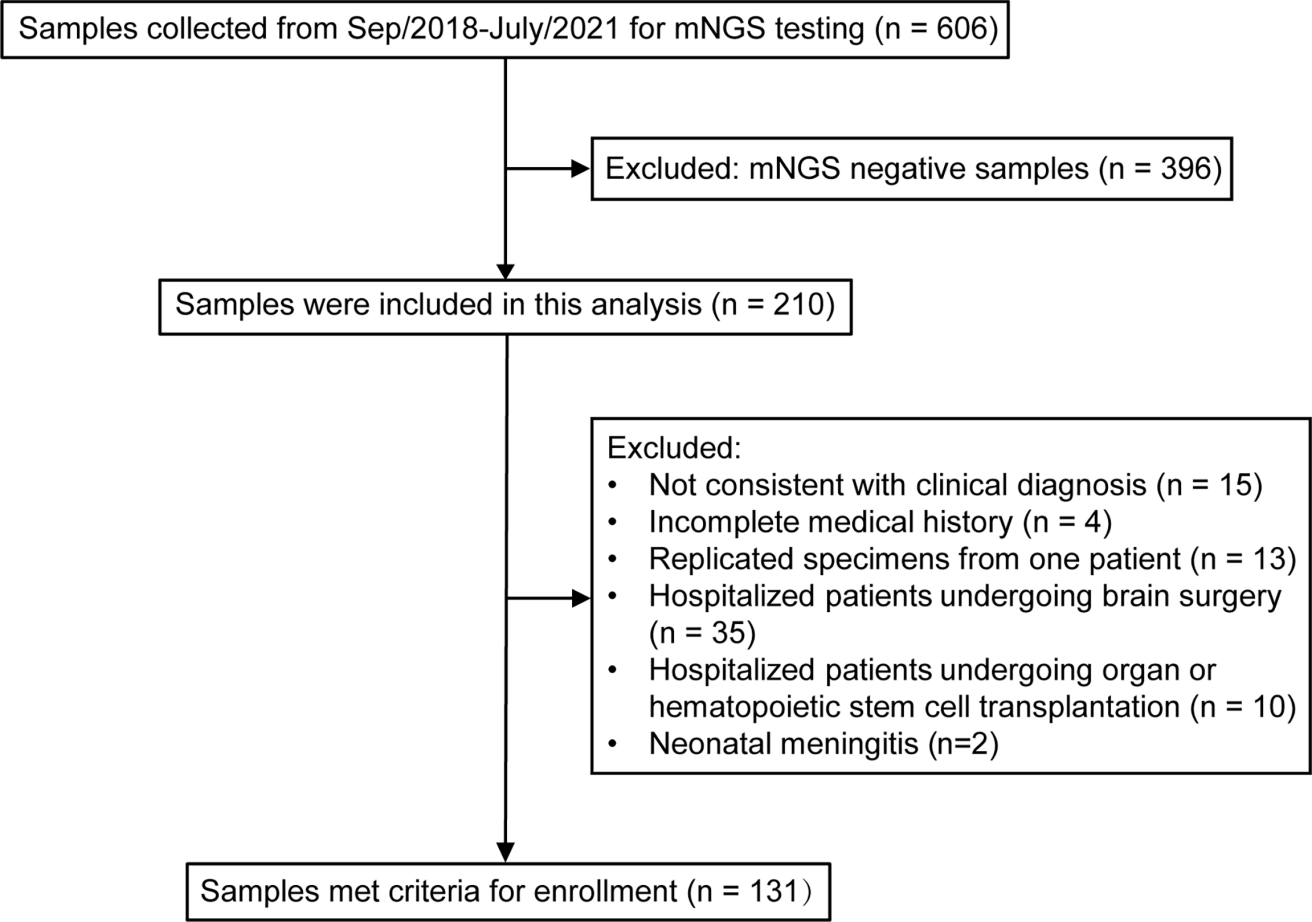
Flowchart of study enrollment.

All patients were admitted to the First Affiliated Hospital of University of Science and Technology of China, which is a large class IIIA general hospital with 5750 beds. From September 2018 to July 2021, suspected CNS infectious patients underwent lumbar puncture after signing informed consents. CSF samples were sent for routine testing, biochemical testing and culture for bacteria and fungi. If the patient was critically ill, the CSF sample was tested by mNGS synchronously; if not, further mNGS was conducted when the CSF culture was negative. Metagenomic transcriptome sequencing was performed when patients were suspected of viral infection but negative for mNGS DNA sequencing. All mNGS-positive samples were included in this analysis, and the exclusion criteria were as follows: not consistent with clinical diagnosis; incomplete medical history; replicated specimens from one patient; hospital-acquired infections (samples from hospitalized patients undergoing brain surgery, organ or hematopoietic stem cell transplantation) and neonatal meningitis. Eligible patients included for analysis were categorized into six groups according to the mNGS results: bacterial infections, viral infections, fungal infections, parasitic infections, other specific pathogen infections and mixed infections. **Figure 1** presents the flow chart of study enrolment.

### DNA extraction, library preparation, and mNGS of CSF

1.5-3 ml CSF sample was collected from each patient by lumbar puncture in accordance with standard procedures. A 1.5 ml microcentrifuge tube with 0.6 ml sample and 250 μl, 0.5 mm glass bead were attached to a horizontal platform on a vortex mixer and agitated vigorously at 2800-3200 rpm for 30 min. Then 7.2 μl lysozyme was added for wall-breaking reaction. 0.3 ml sample was separated into a new 1.5 ml microcentrifuge tube and DNA was extracted using the TIANamp Micro DNA Kit (DP316, Tiangen Biotech) according to the manufacturer’ s recommendation (Long et al., 2016).

Nucleic acids RNA extracted from the clinical samples using the TIANamp Micro RNA Kit (DP431, Tiangen Biotech, Beijing, China) in accordance with the manufacturer’s standard protocols. The reverse transcription reaction was performed to generate single-strand cDNA, followed by the synthesis of double-strand cDNA using the PMseq™ RNA Infection Pathogen High-throughput Detection Kit according to the manufacturer’s instructions.

Then, DNA libraries were constructed through DNA fragmentation, end repair, adapter ligation and PCR amplification. Agilent 2100 was used for quality control of the DNA libraries. Quality qualified libraries were pooled, DNA Nanoball (DNB) was made and sequenced by BGISEQ-50/MGISEQ-2000 platform. Negative control was set as quality control in each test.

### Interpretation of mNGS data

Raw sequence data were first filtered by removing common background microorganisms and low-quality reads. Then the filtered sequences were mapped to the human reference database (hg38) using Burrows-Wheeler alignment to computationally subtract the human sequence (Li and Durbin, 2009). The remaining data were further classified by simultaneously alignment to Pathogens Metagenomics Database (Refseq). The classification reference databases were downloaded from the National Center Biotechnology Information (NCBI) (ftp://ftp.ncbi.nlm.nih.gov/genomes/). RefSeq contains 4945 whole genome sequence of viral taxa, 6350 bacterial genomes or scaffolds, 1064 fungi and 234 parasites related to human infections.

The criteria for positive results were as follows: 1) mNGS identified bacteria (mycobacteria and nocardia excluded), virus and parasites when the coverage rate was 10-fold greater than that of any other microorganisms. 2) mNGS identified Mycobacterium tuberculosis when the genus-specific read number ≥ 1. 3) mNGS identified nontuberculous mycobacteria and nocardia when the mapping read number (genus or species level) was in the top 10 in the bacteria list. 4) mNGS identified fungi when the coverage rate was 5-fold greater than that of any other microorganisms.

### Statistical analysis

Differences across independent binomial variables were evaluated by Chi-square test and Fisher’s exact test. Differences across continuous variables were evaluated by analysis of variance (ANOVA) when they conform to Gaussian distribution and by Mann-Whitney test or Kruskal Wallis test when not. Data analyses were performed using GraphPad Prism software (version 8.0). P values < 0.05 were considered significant, and all tests were two-tailed.

### Standard protocol approvals, registrations, and patient consents

This study was approved by the Ethics Committee of the First Affiliated Hospital of University of Science and Technology of China (approval no. 2021-BE(H)-005). Written informed consent was obtained from all patients or their legal representatives.

## Results

### Clinical characteristics and CSF laboratory examinations of enrolled patients

Between September 2018 and July 2021, a total of 606 samples were collected for mNGS detection, of which 210 were positive. We next excluded 15 samples that not consistent with clinical diagnosis, 4 samples with incomplete medical history, 13 replicated specimens, 45 cases with hospital-acquired infections and 2 cases with neonatal meningitis, and 131 samples were finally enrolled as CA-CNS infections. Overall, males accounted for the majority of patients with bacterial (35/57 [61.40%]) and viral (33/43 [76.74%]) infections, but a minority in the fungal infection group (8/18 [44.44%]). The average age of total 131 patients was 42.37 ± 22.84 years, 18 patients were 12 years of age or younger and 36 patients were over 65 years of age. Bacteria caused more infections in children (11/57 [19.30%]) but fewer infections in the elderly (8/57 [14.03%]) than viruses (children, 9.30%; elderly, 34.89%) and fungi (children, 5.56%; elderly, 22.22%). Bacterial (33/57 [57.89%]) and fungal (12/18 [66.67%]) infections occurred more frequently in spring and summer, while viral (22/43 [51.16%]) infections were more common in fall and winter. The typical neurological manifestations of enrolled patients were headache (66/131 [51.16%]), disturbance of consciousness (47/131 [36.43%]), neck stiffness (30/131 [23.26%]) and convulsions (19/131 [14.73%]), with many patients also presenting systemic symptoms such as fever (94/131 [72.84%]), and vomiting (30/131 [23.26%]). Most patients were admitted to the infectious disease department (42/131 [32.06%]), neurology department (34/131 [25.95%]) or intensive care unit (ICU) (29/131 [22.14%]), followed by the pediatric department (18/131 [13.47%]) or hematology department (6/131 [4.58%]). Critically ill patients who were admitted to the ICU more commonly had bacterial (12/57 [21.05%]) and viral (13/43 [30.23%]) infections than fungal (1/18 [5.56%]) ones. CSF laboratory results in different CNS infections groups revealed that the bacterial subgroup had a significantly higher CSF white blood cell (WBC) count (p < 0.0016) and protein level (p = 0.0053) but a lower glucose level (p = 0.0393) than the viral and fungal subgroups. Besides, CSF chlorine level was significantly lower in fungal infections patients than in those with bacterial and viral infections (p = 0.0037). Thanks to the rapid mNGS diagnosis and timely antibiotic treatment, most infected patients eventually improved or recovered (107 [81.68%]). The clinical and laboratory data are summarized in **Table 1**.

**Table 1:** Clinial characteristics and CSF laboratory examinations of enrolled cases.

	mNGS-positive	Bacterial infections	Viral infections	Fungal infections	p value
	samples (n=131)	(n=57) (a)	(n=43) (b)	(n=18) (c)	among a-c
**Gender no. (%)**					0.0449*
Male	83 (63.36)	35 (61.40)	33 (76.74)	8 (44.44)	
Female	48 (36.64)	22 (38.60)	10 (23.26)	10 (55.56)	
**Age no. (%)**					0.0854
0-12 yr	18 (13.74)	11 (19.30)	4 (9.30)	1 (5.56)	
13-60 yr	77 (58.78)	38 (66.67)	24 (55.81)	13 (72.22)	
>60 yr	36 (27.48)	8 (14.03)	15 (34.89)	4 (22.22)	
**Month no. (%)**					0.4048
Mar.-Aug.	75 (57.25)	33 (57.89)	21 (48.84)	12 (66.67)	
Sept.-Feb.	56 (42.75)	24 (42.11)	22 (51.16)	6 (33.33)	
**Clinical manifestation no. (%)**					
Fever	94 (72.87)	42 (73.68)	31 (75.61)	11 (61.11)	0.5823
Headache	66 (51.16)	32 (56.14)	17 (41.46)	12 (66.67)	0.0996
Neck stiffness	30 (23.26)	19 (33.33)	8 (19.51)	2 (11.11)	0.0843
Vomiting	30 (23.26)	15 (26.32)	9 (21.95)	3 (16.67)	0.648
Disturbance of consciousness	47 (36.43)	19 (33.33)	20 (48.78)	4 (22.22)	0.1579
Convulsions	19 (14.73)	9 (15.79)	8 (19.51)	2 (11.11)	0.7651
**Department no. (%)**					0.0280*
Infectious disease department	42 (32.06)	21 (36.84)	10 (23.26)	7 (38.88)	
Intensive care unit	29 (22.14)	12 (21.05)	13 (30.23)	1 (5.56)	
Neurology department	34 (25.95)	11 (19.30)	14 (32.56)	5 (27.78)	
Pediatric department	18 (13.74)	11 (19.30)	4 (9.30)	2 (11.11)	
Hematology department	6 (4.58)	2 (3.51)	2 (4.65)	1 (5.56)	
Others	2 (1.53)	0	0	2 (11.11)	
**CSF laboratory test,**					
**median (range)**					
CSF WBC (×10^6^/L)	121	211	57	20	< 0.0001*
	(0.00-21229.00)	(1.00-21229.00)	(1.00-579.00)	(1.00-217.00)	
CSF Protein (g/L)	1.03	1.38	0.94	0.49	0.0053*
	(0.20-25.77)	(0.20-25.77)	(0.29-13.61)	(0.22-9.90)	
CSF Glucose (mmol/L)	3.1	2.58	3.32	2.94	0.0393*
	(0.05-14.13)	(0.15-7.19)	(0.83-7.61)	(1.32-4.42)	
CSF Chlorine (mmol/L)	118.3	118.3	117.4	104.9	0.0037*
	(94.70-137.70)	(94.70-137.70)	(106.4-132.20)	(116.00-132.80)	
**Outcome no. (%)**					0.7245
Improvement	107 (81.68)	45 (78.95)	37 (86.05)	16 (88.89)	
Progression	21 (16.03)	10 (17.54)	6 (13.95)	2 (11.11)	
Death	3 (2.29)	2 (3.51)	0	0	

Statistics: Chi-square or Fisher’s exact test for calculations of clinical characteristics. ANOVA or Kruskal Wallis test for calculations of CSF laboratory examinations. *P value < 0.05.

### Etiological diagnosis of CA-CNS infections using mNGS

**Figure 2:**
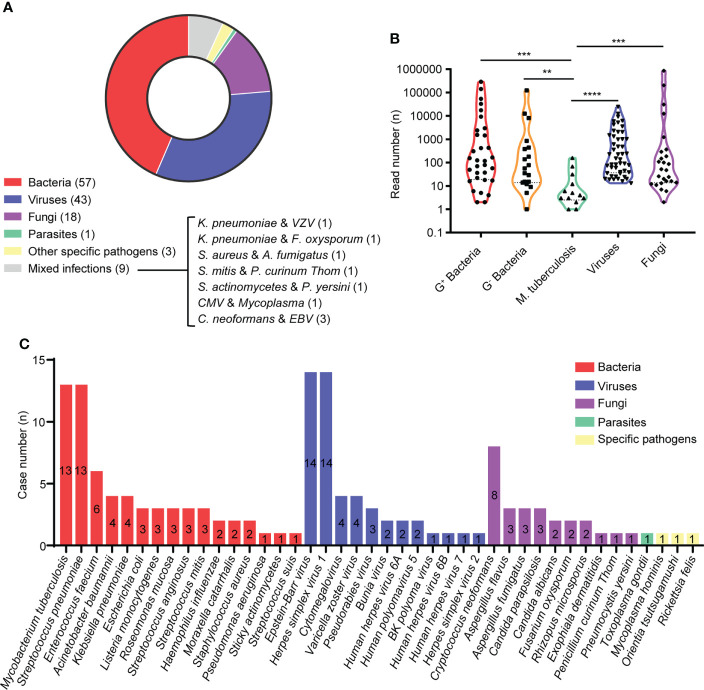
Etilolgy of CA-CNS infections identified by mNGS in the present study. **(A)** Pie charts demonstrated the distribution of different types of microbial infections. **(B)** Violin polot showed read numbrers of pathogen species. G^+^ bacteria (n = 32); G^-^ bacteria (n = 19); *Mycobacterium tuberculosis* (n = 13); viruses (n = 49); fungi (n = 26). Data were analyzed by Mann-Whitney test. **(C)** A total of 40 different species of pathogens were detected in the different infectious groups with their corresponding frequencies plotted in histograms. ** p < 0.01, *** p < 0.001, **** p < 0.0001.

In this study, etiological diagnosis showed that bacterial infections (57/131 [43.51%]) were the most common, while viral, fungal, parasitic and specific pathogen infections were accounted for 32.82% (43/131), 13.74% (18/131), 0.76% (1/131) and 2.29% (3/131) of all patients, respectively. In 9 (6.87%) patients, two causative agents were identified, including 3 with recorded mixed infections of Cryptococcus neoformans and Epstein-Barr virus (**Figure 2A**). In addition, we found the positive rate of was 7.63% (10/131) by culture, 21.37% (28/131) by antigen test and 3.05% (4/131) by PCR, including 4 were confirmed by two different methods (**Supplementary Table**). The read numbers of Mycobacterium tuberculosis were significantly lower than those of other types of microbes, while these cases were eventually confirmed as tubercular meningitis (TBM) through the medical history, other examination results and treatment outcomes (**Figure 2B**).

As shown in **Figure 2C**, we identified 140 pathogens of 41 different pathogens in the CSF from 131 patients. 16 different agents were detected from patients with bacterial infections, of which Mycobacterium tuberculosis (13 cases) and Streptococcus pneumoniae (13 cases) were the most frequent causative microbes, followed by Enterococcus faecium (6 cases), Acinetobacter baumannii (4 cases) and Klebsiella pneumoniae (4 cases). Among viral infections caused by 13 different agents, the top 5 most commonly detected pathogens were Epstein-Barr virus (EBV) (14 cases), Herpes simplex virus 1 (HSV1) (14 cases), Cytomegalovirus (CMV) (4 cases), Varicella zoster virus (VZV) (4 cases) and Pseudorabies virus (PRV) (3 cases), while 2 Bunyavirus infections were observed using RNA mNGS. The most frequently detected fungal infections were cryptococcal meningitis (8 cases), aspergillus meningitis (6 cases) and candidal meningitis (5 cases). In addition, 1 case was parasitic infection caused by Toxoplasma gondii and 3 cases were infected with other specific pathogens [Mycoplasma hominis (n = 1), Orientia tsutsugamushi (n = 1), Rickettsia (n = 1)].

### Diagnosis of CA-CNS infections caused by specific pathogens using mNGS

mNGS of has already been widely used for identifying novel or unexpected pathogens in various infectious diseases, we therefore report herein the utility of mNGS for identifying microbial pathogens in five challenging cases of CA-CNS infections in our study.

#### Streptococcus suis

This patient was a 59-year-old butcher. On September 2018, he hurt his left finger when dissecting dead swine. Two days later, he presented with vomiting, fever, weakness and somnolence, which rapidly progressed to coma and petechiae and was admitted to the ICU. His WBC count was 17.04 × 109/L with 88.7% neutrophils; prothrombin time (PT) was 48.8 s; D dimer (D-D) > 20.00 mg/L; procalcitonin (PCT) was 100 ng/ml and C-reactive protein (CRP) was 107.98 mg/L. CSF examination revealed a WBC count of 233 × 109/L comprising 62% neutrophils, a glucose level of 0.5 mmol/L, a total protein level of 2.6 g/L and a chlorine level of 120.0 mmol/L. Bacterial and fungal cultures of blood and CSF were negative, raising concern for an alternative diagnosis. His CSF mNGS data contained 289518 reads aligning to Streptococcus suis. After etiological diagnosis was achieved, he was treated with a high dose of penicillin for 16 days and repeated blood and CSF examination results improved to the baseline.

#### Pseudorabies virus

**Figure 3:**
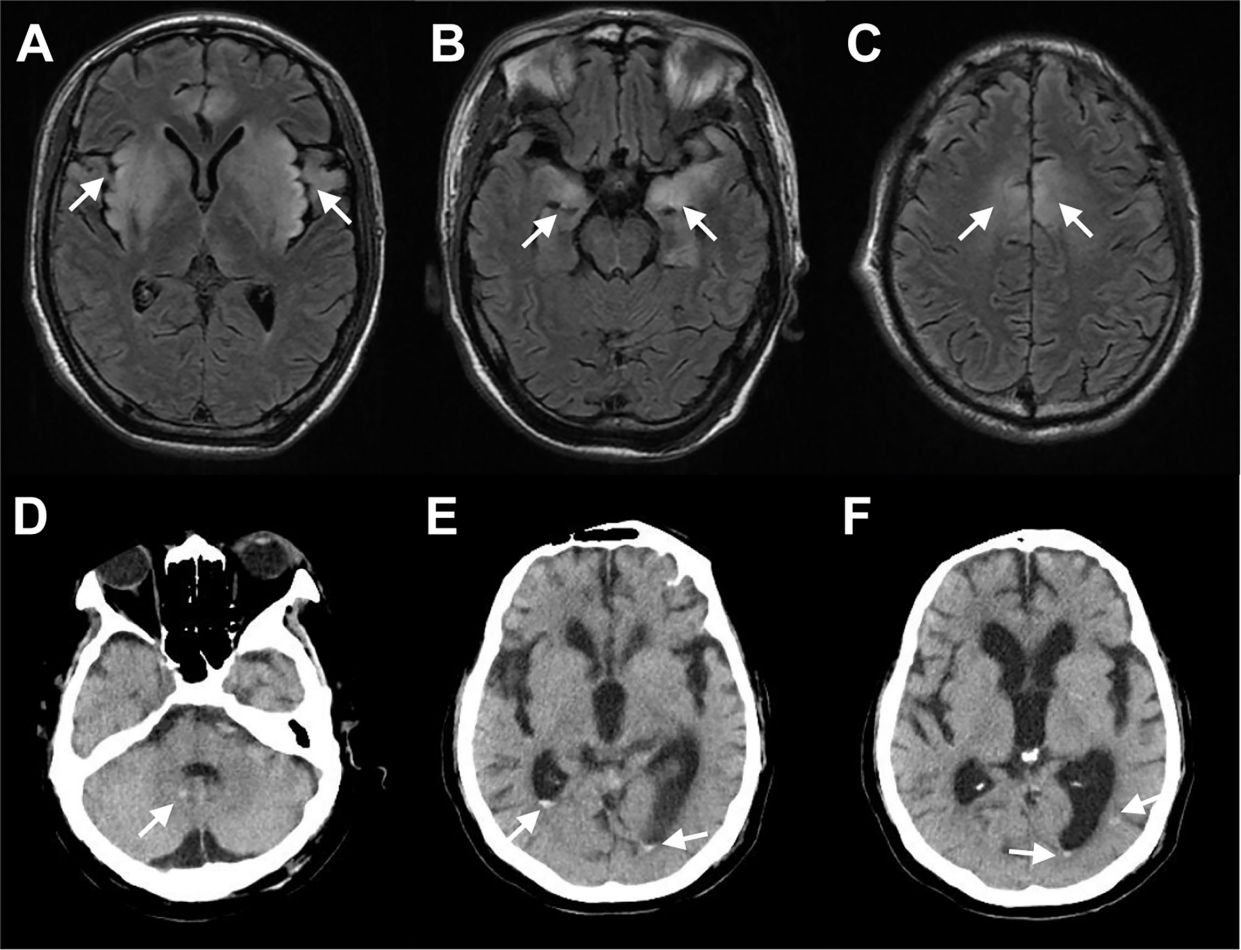
Selected Neuroimaging. **(A–C)** MRI of patient infected with *Porcine herpesvirus 1* showed hyperintense signal distributed symmetrically in the bilateral insula lobe, the basal ganglia, the hippocampus, and the frontal lobe on T2 fluid-attenuated inversion recovery (FLAIR). **(D–F)** CT of patient infected with *Toxoplasma gondii* showed multiple oval high-density shadows in the right cerebellar hemisphere, the posterior regions of bilateral ventricles, and the posterior corner of the left lateral ventricle. Arrows point to areas with enhanced signals.

A previously healthy 44-year-old pork vender was admitted to our hospital on January 2019 with a 4-day history of headache and cough and a 1-day history of fever (41°C) and intermittent convulsions. Other symptoms included neck stiffness and disturbance of consciousness. Routine laboratory investigations revealed CRP was 18.40 mg/L; Creatine kinase (CK) was 16111 IU/L; CK-MB was 150 IU/L and Myoglobin (Mb) was 8770 ng/ml. The rest of the blood and CSF laboratory tests were all within the normal range. Magnetic resonance imaging (MRI) of the brain showed hyperintense signal distributed symmetrically in the bilateral cerebral hemispheres (**Figures 3–C**), which was consistent with the characteristics of encephalitis. Etiology diagnoses including serum TORCH series, immune combination and PCR of EBV and CMV were all negative. We treated him with sodium valproate to prevent seizures and sent the CSF samples for analysis with mNGS. The results showed 83 reads specifically mapped to Pseudorabies virus. Then, the CSF specimen sent for PCR amplified PRV and the result of CSF PRV IgG antibody test was positive. After 2 months of treatment with acyclovir, ceftriaxone and sodium valproate, he showed mild clinical improvement and was transferred to the rehabilitation hospital.

#### Bunyavirus

On March 2021, a 40-year-old woman was bitten by insects when picking tea and then presented with diarrhea, fatigue and fever (39°C). She was admitted to the infectious disease department and laboratory tests showed WBC count was 1.99 × 109/L with 61.6% neutrophils; and platelet (PLT) count was 38 × 109/L. CSF routine and biochemistry tests were within the normal ranges. Serum TORCH series and antibody tests for EBV and tsutsugamushi disease were negative, while Hepatitis B virus (HBV) antigen test was positive. The patient was treated empirically with ribavirin, minocycline and levofloxacin. However, she developed worsening unconsciousness and coagulation disorders, with gradual decreases of blood pressure (67/26 mmHg) and oxygen saturation (SaO2) (78%). Then she was transferred to the ICU and CSF sample was immediately tested by mNGS. The sequencing detection identified 1321 RNA sequence reads uniquely corresponding to Bunyavirus. The patient was subsequently treated with continuous blood purification combined with antiviral therapy. Nevertheless, the disease rapidly progressed with aggravated shock and disseminated intravascular coagulation (DIC), and she was finally discharged voluntarily.

#### Orientia tsutsugamushi

On November 2020, a 71-year-old woman was admitted to the neurology department of our hospital because of fever and insanity without obvious predisposing factors for 5 days. She presented with intermittent convulsions, unable to recognize her family, raving and urinary incontinence. Upon admission, PLT count was 87 × 109/L and CRP was 298.6 mg/L. The rest of the blood laboratory tests were all within the normal range. The cytology of CSF demonstrated increased WBCs (23 × 106/L) and protein concentration (0.78 g/L). After admission, the patient’s symptoms gradually deteriorated with progressive decrease of PLT count, myocardial damage and pulmonary infection. Bacterial cultures were negative while CSF mNGS returned 14 reads mapping to Orientia tsutsugamushi. After etiological diagnosis was identified, treatment with levofloxacin and mannitol was initiated. On the 20th day, her clinical condition was noticeably improved with recovered from fever and pulmonary infection. A repeat lumbar puncture revealed improved WBCs and protein. Thus, she was discharged on the 23rd day.

#### Toxoplasma gondii

A 68-year-old male patient was admitted to our hospital on October 2019 due to fever (40°C) accompanied by diarrhea for 3 days. He had a history of coronary heart disease for 4 years and had received heart transplant 1 month previously. After the surgery, he had been taking immunosuppressive drugs including tacrolimus, motimecocuronol ester and hormone. Blood tests on admission showed WBC count of 13.4 × 109/L, neutrophil rate of 82.6%; CRP of 62.27 mg/L; CK of 564.0 IU/L; CK-MB of 249 IU/L; and brain natriuretic peptide (BNP) of 3734 pg/ml. Lumbar puncture was subsequently performed and CSF WBC count was 307 × 106/L, of which neutrophils accounted for 94%; proteins was 1.50 g/L; glucose was 14.13 mmol/L; chlorine was 119 mmol/L. Brian computed tomography (CT) revealed multiple oval high-density shadows in the right cerebellar hemisphere and the posterior regions of bilateral ventricles (**Figure 3D–F**). After empirical treatment with ceftizoxime, azithromycin and oseltamivir, his symptoms worsened with the developed oliguria, dyspnea and decreased SaO2. His CSF was collected for mNGS to determine the pathogen and the results showed a total of 8999 unique sequences of Toxoplasma gondii. CSF specimen that had been subsequently sent for PCR also amplified the Toxoplasma gene. After the diagnosis was confirmed, sulfonamides were replaced for anti-infection treatment. However, the patient’s condition aggravated progressively and he eventually died.

## Discussion

The clinical management of patients with CA-CNS infections is highly dependent on the underlying cause of infection or inflammation, making it necessary to obtain a specific etiological diagnosis whenever possible (Polage and Cohen, 2016; van de Beek et al., 2016). Culture remains the mainstay in diagnosing CNS infections, while in clinical laboratories, culture is only used for bacteria and fungi detection. Viruses can grow and reproduce only by obligate parasitism in living sensitive host cells, so the culture medium of viruses is demanding, highly specific, and the culture processes are too complex to be available in many clinical laboratories (Storch, 2000; Kelley and Caliendo, 2001). In our study, mNGS detected 56 cases (42.75% [56/131]) infected with viruses, parasites and other specific pathogens that cannot be cultured. Actually, owing to the use of antibiotics before lumbar puncture and the slow growth of some pathogens such as Mycobacterium tuberculosis and Cryptococcus neoformans, the yield of CSF cultures in suspected cases is low (Glaser et al., 2006). Zhang et al. reported that in 83 cases of mNGS positive CSF specimens, the culture positive rate was 18.07% (15/83) (Zhang et al., 2020). In our study, mNGS was selected only when the patient was critically ill or CSF culture was negative, so the positive rate of culture was only 7.63% (10/131) in the mNGS-positive population.

FilmArray meningitis/encephalitis panel is a multiplex PCR assay from BioFire Diagnostics to simultaneously detects 14 pathogens, including six bacteria (S. pneumoniae, N. meningitidis, H. influenzae, S. agalactiae, E. coli K1, and L. monocytogenes), seven viruses (enterovirus, HSV-1/2, VZV, CMV, HHV-6, and human parechovirus), and C. neoformans/C. gattii from ≤ 200 μl of CSF in about 1 h (Leber et al., 2018; Tansarli and Chapin, 2020). A total of 42 different species of pathogens were identified from 131 cases in this study and 32 of them from 55 cases were not included in FilmArray panel, indicating that this method is unable to detect all microbes in general and cannot meet the need for diagnosing CA-CNS infections.

Since Wilson et al. identified Leptospira santarosai in the CSF sample of a case with meningoencephalitis in 2014 (Wilson et al., 2014), there has been great interests in the use of mNGS for pathogen detection (Goldberg et al., 2015; Miao et al., 2018; Miller et al., 2019). Miao et al. reported that mNGS could yield a higher sensitivity for pathogen identification and is less affected by prior antibiotic exposure (Miao et al., 2018). In 2021, mNGS was recommended in an expert consensus to assist clinical decision making in cases with CNS infections (Chinese Society of Infectious Diseases and Cerebrospinal Fluid Cytology, 2021). Thus, we sought to define the performance of mNGS testing in CA-CNS infections, especially in the difficult-to-diagnose patient population.

Bacterial meningitis is a considerable burden worldwide. It has been reported that Streptococcus pneumoniae, Neisseria meningitidis, Haemophilus influenzae, and Listeria monocytogenes are the most common pathogens of community-acquired suppurative meningitis (Bijlsma et al., 2016). In our study, there were 13 cases of Streptococcus pneumoniae, 4 of Haemophilus influenzae and 3 of Listeria monocytogenes, while no Neisseria meningitidis was found, which may be related to the vaccination in China. Among the 13 cases of Streptococcus pneumoniae infection, 6 involved were children and 2 finally led to progression or death, demonstrating the importance of pneumococcal immunization (McGill et al., 2016). Listeria monocytogenes is an uncommon but important cause of severe CA-CNS infections. However, it is a challenge for clinicians to diagnose listeria encephalitis at an early stage because of the low positive rate by Gram staining and culture (Paul et al., 1994). In this study, we identified 3 cases of Listeria monocytogenes, highlighting the feasibility of mNGS as a diagnostic method for CNS listeria infections. In addition, we found TBM accounted for 9.92% (13/131) of CA-CNS infections. Xing et al. reported that among the 44 patients with presumed TBM, CSF culture were all negative and Xpert MTB/RIF positivity rate was 16.13% (5/31), while mNGS positivity rate was 27.27% (12/44) (read number ≥ 1), indicating mNGS is extremely feasible for diagnosing TBM when at least one specific read is matched to the Mycobacterium tuberculosis complex (Xing et al., 2020).

We found that viruses are the second most common pathogens after bacteria, and 5 patients had mixed infections with bacteria or fungi. Actually, in clinical work, viral and/or mixed infections are easily ignored, but mNGS can diagnose them more effectively. EBV is the most frequent causative virus in our cohort and 3 cases were co-infected with Cryptococcus neoformances. The clinical significance of EBV in CSF is not completely understood. Kelly et al. reported that immunosuppressed patients have elevated concentrations of EBV antibodies in the blood due to large numbers of EBV-infected B lymphocytes, and high EBV viral load is a marker of poor outcome in individuals with co-infections (Kelly et al., 2012). In this study, we also observed RNA sequence reads corresponding to Bunyavirus in 2 cases. It is now possible to detect RNA viruses using mNGS, but the process is complex and expensive, so it is not used in all cases.

Our data showed that the pathogens of fungal infections are widely distributed and accounted for the largest proportion of co-infected patients, among which Cryptococcus neoformans is the most common. Cryptococcus neoformans is also the most common cause of adult meningitis in HIV-infected individuals. It is also found in an increasing number of patients with other types of natural and iatrogenic immunosuppression (Williamson et al., 2017). Among the 8 patients infected with Cryptococcus neoformans in this study, 4 had rheumatic autoimmune disease and 3 had mixed infection. In addition, only one Cryptococcus neoformans was detected by culture in all samples, indicating mNGS has advantages in diagnosis of fungal infections. A descriptive study also showed that mNGS was regarded as a useful tool for the identification of cryptococcal meningitis (Xing et al., 2019).

We further selected 5 challenging cases of CA-CNS infections caused respectively by Streptococcus suis, Pseudorabies virus, Bunyavirus, Orientia tsutsugamushi and Toxoplasma gondii that were confirmed using mNGS. Streptococcus suis and Porcine herpesvirus 1 are zoonotic pathogens that can cause cross-species transmission and induce human infections (Wang et al., 2018; Tan et al., 2021). Most patients have occupations related to pig farming or butchery and become infected via the invasion of wounds by pathogens. Bunyavirus and Orientia tsutsugamushi are insect-borne pathogens which are transmitted to humans through the bites of infected insects. Bunyavirus infection often occurs in environments inhabited by ticks between March and October. Most patients have a history of working in mountains and presented with multiorgan dysfunction (Hulswit et al., 2021). Oriental tsutsugamushi infection, also named as scrub typhus, has clear regional and seasonal characteristics, often occurring in southern China between May and November. Rodents are the main source of this infection, which is transmitted to humans through chigger bites. The toxin released after death of Orientia tsutsugamushi causes toxemia and multiorgan lesions (Lu et al., 2021). The patient who infected with Toxoplasma gondii in this study had received heart transplant and taken immunosuppressants for a long time, making him are susceptible to toxoplasma infection. Toxoplasmic encephalitis is an opportunistic CNS infectious disease caused by the zoonotic pathogens Toxoplasma gondii. It is more common to occur in patients with immunodeficiency or long-term immunosuppressive therapy. With the continuous renewal of immunosuppressive agents in recent years, the clinical incidence of toxoplasmic encephalitis has increased significantly (Kochanowsky and Koshy, 2018). Infections with these pathogens often cause acute symptoms and are difficult to be detected by conventional methods. Therefore, mNGS is an accurate and time-saving technology for the diagnosis of infectious diseases in these conditions.

There are some limitations in our study. Firstly, our data are from a single-center study and cannot fully reflect the etiology of CA-CNS infections. Furthermore, RNA library preparations were conducted in a limited number of patients suspected of viral infection but negative for mNGS DNA sequencing, which might let to neglection of some neuroinvasive RNA viruses like enterovirus. Finally, this is a retrospective study without a careful design in the early stage. Therefore, the detection methods used in different cases were not completely consistent. For example, mNGS and culture were conducted synchronously only when the patient was critically ill, while in other cases mNGS was conducted when the culture was negative so that the positivity rate of culture was extremely low in this study. In Conclusion, CA-CNS infections are caused by a wide variety of pathogens so that traditional methods cannot meet the diagnostic requirements. Our data provided a better understanding on the etiology of CA-CNS infections and showed that mNGS represents a comparative screening of CSF in an unbiased manner for a broad range of human pathogens.

## Data availability statement

The raw sequence data reported in this paper have been deposited in the Genome Sequence Archive in National Genomics Data Center, China National Center for Bioinformation / Beijing Institute of Genomics, Chinese Academy of Sciences (GSA: CRA008203) that are publicly accessible at https://ngdc.cncb.ac.cn/gsa.

## Ethics statement

The studies involving human participants were reviewed and approved by Ethics Committee of the First Affiliated Hospital of University of Science and Technology of China (USTC) (approval no. 2021-BE(H)-005). Written informed consent to participate in this study was provided by the participants’ legal guardian/next of kin. Written informed consent was obtained from the individual(s) for the publication of any potentially identifiable images or data included in this article.

## Author contributions

SZ and GW were responsible for data interpretation, figure preparation, and manuscript drafting and editing; TYS and TL were responsible for next-generation sequencing analysis for this study. LX, YD and WC were responsible for acquisition of clinical data and manuscript editing. XM were responsible for overall project conception, study design, data interpretation and manuscript preparation and editing. All authors contributed to the article and approved the submitted version.

## Acknowledgments

We thank the patients for cooperating with our investigation and acknowledge the professionalism and compassion demonstrated by all the healthcare workers involved in patients’ care.

## Conflict of interest

The authors declare that the research was conducted in the absence of any commercial or financial relationships that could be construed as a potential conflict of interest.

## Publisher’s note

All claims expressed in this article are solely those of the authors and do not necessarily represent those of their affiliated organizations, or those of the publisher, the editors and the reviewers. Any product that may be evaluated in this article, or claim that may be made by its manufacturer, is not guaranteed or endorsed by the publisher.
